# Demographics, Tobacco Use, and Tobacco Control Measures of California Cities With Flavored Tobacco Sales Restrictions

**DOI:** 10.1177/15248399221136861

**Published:** 2022-11-23

**Authors:** Melanie S. Dove, Shichen Zheng, Sheila Pakdaman, Julia Chen-Sankey

**Affiliations:** 1 Department of Public Health Sciences, University of California, Davis, Davis, CA, USA; 2 Department of Population and Public Health Sciences, University of Southern California, Los Angeles, CA, USA; 3 Rutgers Biomedical and Health Sciences, New Brunswick, NJ, USA; 4 Rutgers School of Public Health, Piscataway, NJ, USA

**Keywords:** tobacco control, youth, e-cigarette, flavors, policy, disparities, data linkage

## Abstract

In 2020, California passed a flavored tobacco sales restriction (FTSR), but the tobacco companies filed a referendum, and the ban will not be implemented unless approved by voters in November 2022. This study examined the percentage of the California population covered by a city FTSR and identified groups more likely to be covered. Mean demographics as well as tobacco use and control measures were compared for California cities with (*n* = 93) and without (*n* = 389) a FTSR, and t tests were used to examine the differences. We calculated adjusted odds ratios using logistic regression models. City FTSR policies covered 20.7% of the California population. Adjusted predictors of having a FTSR included the American Lung Association tobacco control score (odds ratio [OR] = 1.27, 95% confidence interval [CI]: [1.17, 1.38]), voting democratic (OR = 1.06, 95% CI: [1.02, 1.10]). and having a lower adult smoking prevalence (OR = 0.84, 95% CI: [0.72, 0.99]). A state-level policy would cover all populations in California.

E-cigarettes are the most used tobacco product among high school students, with 11.3% currently using in 2021 (Gentzke et al., 2022). Almost all youth (84.7%) who currently used e-cigarettes used a flavored product and almost half of current youth cigar smokers used flavored cigars (Gentzke et al., 2022). Flavors in tobacco products have been found to contribute to curiosity and interest toward tobacco use, product initiation, long-term and increased use as well as difficulty quitting (Boyle et al., 2019; Persoskie et al., 2016; Sterling et al., 2015).

Flavored tobacco sales restrictions (FTSRs) have the potential to reduce youth use of flavored tobacco products. Studies show that local FTSRs decreased the availability and sales of flavored tobacco products and were associated with a decrease in youth and young adult tobacco use (Rogers et al., 2021). As of March 2022, almost 20% of the U.S. population was covered by an FTSR (Truth Initiative, 2021). However, certain population groups were less likely to be covered by an FTSR including, youth, American Indian/Alaskan Native, and Native Hawaiians/Pacific Islander populations, which is similar to other tobacco control policies (Rose et al., 2020).

The percentage of the California population covered by an FTSR (22.1%) is similar to the national percentage (20.0%; California Department of Public Health, California Tobacco Control Program, 2021). In August 2020, California passed a state-level FTSR (Senate Bill 793), but the tobacco companies filed a referendum, and the ban will not be implemented unless approved by voters in the November 2022 general election (Public Health Law Center, 2020). This study examines demographic and tobacco control measures associated with passing a local FTSR in California and considers how the state policy may reduce any disparities in coverage.

## Method

### Flavored Tobacco Sales Restrictions

FTSRs at the city level passed before September 2021 were identified from national organizations that tracked FTSRs including the Truth Initiative (2021), Campaign for Tobacco-Free Kids (2021), and the Flavored Tobacco Policy Evaluation Tracking System, a database maintained by the American Non-Smokers Rights Foundation and California Tobacco Control Program. The Public Health Law Center also provided a list of local policies and was consulted for any questions regarding policy details. Supplemental Table 1 shows a list of the 93 cities identified as having a policy. County-level policies were not included because they only applied to unincorporated areas of the county, which presented a challenge to merge with other data and represents a smaller proportion of the population.

### Covariates

#### City-Level

Because most FTSRs were implemented during or after 2019 (79 out of 93), we included data on covariates during or prior to 2019 to examine pre-policy characteristics. Information on city-level covariates included population size and percent of the population with less than or equal to a high school education, below the poverty level, aged 15–19, and in any of the racial/ethnic categories (any American Indian or Alaskan Native, any Pacific Islander or Native Hawaiian, any Asian, Any Black, any Hispanic or Latino, any Other, and any White) and was obtained from the 2015–2019 American Community Survey. Information on the number of tobacco retailers within 1,000 feet of a school came from the California Department of Tax and Fee Administration 2018 (California Tobacco Health Assessment Tool, 2020). The 2018 overall tobacco control score (scale 0–12) came from the American Lung Association. This is a sum of three categories: smoke-free outdoor air, smoke-free housing, and reducing sales of tobacco products (based on the strength of their local tobacco retailer licensing ordinance), plus emerging issue bonus points (including 1 point for prohibiting the sale of flavored tobacco products; American Lung Association, 2018). One city (Monte Sereno with a population of 3,479) was missing from the overall tobacco control score. The percentage of high school students who used e-cigarettes was obtained from the 2017/2018–2018/2019 California Healthy Kids Survey (CHKS). Cities with less than 10 students were not included. The CHKS does not include all cities and we were able to merge 324 out of 482 cities.

#### County-Level

Additional covariates merged by county included the percentage of stores that sold a flavored tobacco product other than cigarettes (Healthy Stores for a Healthy Community, 2016), the percentage of the county that voted democratic in the 2016 presidential election (California Presidential Election Results, 2016), the percentage of the county classified as rural (U.S. Census Bureau, 2012), and the percentage of adults who smoked cigarettes (2016–2018 California Health Interview Survey). In addition to data at the county level, Healthy Stores for a Healthy Community included three cities with their own health department (Berkeley, Pasadena, and Long Beach) that were included in our analysis (Healthy Stores for a Healthy Community, 2016).

### Data Analysis

We calculated the mean of each variable overall and among cities with and without FTSRs. The *t* tests were used to examine differences in characteristics between cities with and without an FTSR. To examine which predictors were associated with having an FTSR, we calculated adjusted odds ratios using logistic regression models. The first model adjusted for all variables except for the percentage of high school students who used e-cigarettes because these data were only available for a subset of cities (324 out of 482). The second model additionally adjusted for the percentage of high school students using e-cigarettes. Statistical analysis was performed using SAS version 9.4 (SAS Institute, Cary, NC).

## Results

A total of 93 cities covering 20.7% (*n* = 6,785,621) of the population had an FTSR (Table 1). Compared to cities without an FTSR, cities with an FTSR had a lower mean percentage aged 15–19, percentage of the population living below the poverty level, percentage with less than a high school education, percentage rural, percentage Hispanic or Latino, and percentage other race/ethnicity. Cities with an FTSR had a higher mean percentage that voted democratic in the 2016 election and percentage Asian.

**Table 1:** Characteristics of Cities in California and Association With Flavored Tobacco Sales Restrictions

	*Flavored tobacco sales restriction*					
Characteristics	*Total*	*Yes*	*No*	*p^c^*	*Adjusted OR* ^a^ *[95% CI], n = 481*	*Adjusted OR* ^b^ *[95% CI], n = 323*
	*M (SE)*	*M (SE)*	*M (SE)*			
Number of cities (percent of cities)	482	93 (19.3%)	389 (80.7%)			
Population number (percent of population)	32,763,161	6,785,621 (20.7%)	25,977,540 (79.3%)			
Demographic variables						
Total population	67,973.4 (9,505.9)	72,963.7 (12,560.8)	66,780.3 (11,394.8)	.80	0.95 [0.57, 1.60]	0.84 [0.47, 1.52]
Percentage 15–19 years	6.4% (0.08)	6.0% (0.21)	6.5% (0.09)	.04	1.01 [0.86, 1.19]	1.03 [0.83, 1.29]
Percentage below poverty	13.1% (0.37)	10.5% (0.7)	13.8% (0.42)	<.001	1.02 [0.96, 1.08]	0.98 [0.91, 1.06]
Percentage with ≤ to a high school education	37.2% (0.87)	28.2% (1.92)	39.4% (0.94)	<.001	1.03 [0.98, 1.08]	1.04 [0.99, 1.07]
Percentage of county that is rural	9.9% (0.71)	5.7% (1.01)	10.9% (0.83)	.004	1.01 [0.98, 1.05]	1.02 [0.98, 1.07]
Percentage of county that voted democratic	**58.9% (0.61)**	**68.0% (1.26)**	**56.8% (0.64)**	**<.001**	**1.06 [1.02, 1.10]**	1.04 [0.99, 1.09]
Percentage in each race/ethnic group						
Any American Indian or Alaska Native	2.2% (0.10)	1.8% (0.14)	2.3% (0.12)	.07	1.03 [0.86, 1.23]	1.09 [0.86, 1.38]
Any Native Hawaiian or Other Pacific Islander	0.72% (0.03)	0.81% (0.09)	0.7% (0.04)	.21	1.02 [0.70, 1.47]	0.89 [0.54, 1.45]
Any Asian	13.8% (0.66)	17.4% (1.60)	12.9% (0.72)	.007	1.06 [0.90, 1.25]	1.05 [0.84, 1.32]
Any Black or African American	4.9% (0.25)	5.3% (0.64)	4.8% (0.27)	.39	1.09 [0.93, 1.28]	1.05 [0.84, 1.32]
Any Hispanic or Latino	37% (1.17)	26.7% (2.40)	39.5% (1.30)	<.001	0.99 [0.95, 1.02]	0.97 [0.93, 1.02]
Any other	12.6% (0.55)	9.2% (0.93)	13.4% (0.64)	.003	1.03 [0.86, 1.22]	1.00 [0.79, 1.26]
Any White or European American	71.1% (0.85)	71.2% (2.08)	71.0% (0.93)	.95	1.08 [0.92, 1.27]	1.06 [0.85, 1.32]
Tobacco use and control measures						
Percentage of stores selling flavored tobacco products	81.6% (0.27)	80.1% (0.70)	82.0% (0.29)	.006	1.04 [0.98, 1.10]	1.04 [0.98, 1.11]
Number of tobacco retailers within 1,000’ of a school	16.5 (3.45)	17.9 (4.36)	16.1 (4.15)	.84	1.00 [0.99, 1.02]	1.00 [0.99, 1,02]
American Lung Association tobacco control score	**3.4 (0.17)**	**6.9 (0.41)**	**2.6 (0.16)**	**<.001**	**1.27 [1.17, 1.38]**	**1.34 [1.21, 1.50]**
Percentage of adults in the county who smoke cigarettes	**11.8% (0.16)**	**10.0% (0.32)**	**12.3% (0.18)**	**<.001**	**0.84 [0.72, 0.99]**	**0.83 [0.70, 0.99]**
Percentage of high school students who use e-cigarettes	12.7% (0.33)	14.2% (0.82)	12.3% (0.35)	.01	—	0.97 [0.90, 1.05]

*Note*. OR = odds ratio; CI = confidence interval. Bold values indicate statistically significant (*p* < .05) adjusted odds ratios.

a Adjusted for total population (divided by 100,000), percentage 15–19 years, percentage below poverty, percentage with less than a high school education, percentage of the county that was rural, percentage of the county that voted democratic in 2016, percentage American Indian/Alaskan Native, percentage Pacific Islander or Native Hawaiian, percentage Asian, percentage Black, percentage Hispanic or Latino, percentage other race, percentage of stores in the county selling flavored tobacco products, number of retailers near schools, tobacco control score, and percent of adult smokers in the county. ^b^Additionally adjusted for the percent of high school students using e-cigarettes. ^c^*p* value from *t* test comparing mean.

Cities with FTSRs had evidence of stronger tobacco control efforts. Compared with cities without an FTSR, cities with an FTSR had a lower percentage of stores selling tobacco products and a higher tobacco control score. Although cities with an FTSR had a lower adult smoking prevalence, they had a higher prevalence of high school students who currently used e-cigarettes. There was no difference in the number of tobacco retailers near schools between the two groups.

Adjusted for covariates in the first model, the strongest predictor of having an FTSR was the tobacco control score (odds ratio [OR] = 1.27, 95% confidence interval [CI]: [1.17, 1.38]), followed by the percent that voted for the democratic candidate in the 2016 presidential election (OR = 1.06, 95% CI: [1.02, 1.10]) and the percent of the population who smoked cigarettes (OR = 0.84, 95% CI: [0.72, 0.99]). Adding high school e-cigarette use to the model did not change these estimates substantially and was not associated with having an FTSR.

## Discussion

There were 93 local FTSRs implemented before September 2021 in California, covering approximately 20% of the population. Although there were unadjusted differences in cities with and without an FTSR by age, poverty, education, and race/ethnicity, the strongest predictors of having a city FTSR were higher tobacco control scores and lower cigarette smoking prevalence. These results suggest that FTSRs were implemented in cities that already had a strong record of tobacco control policies and less cigarette use. This may leave the localities that are already vulnerable to tobacco use further unprotected from the widespread marketing and sales of flavored tobacco products.

Our findings that approximately 20% of the California population was covered by an FTSR are consistent with an analysis conducted by the California Tobacco Control Program, which found 22.1% coverage (California Department of Public Health, California Tobacco Control Program, 2021). They also found that Hispanic or Latino persons were less likely to be covered by FTSRs than their non-Hispanic White counterparts (California Department of Public Health, California Tobacco Control Program, 2021). Efforts in restricting the sales of flavored tobacco should be further made in those vulnerable communities to reduce health disparities.

There were other notable differences between cities with and without an FTSR. Cities with an FTSR had a slightly lower mean percent of youth aged 15–19 compared with cities without an FTSR. As this age group has the highest prevalence of flavored tobacco use, tobacco control efforts, such as policies and cessation tools, should target this age group. In addition, cities with an FTSR had a lower mean percentage rural and a higher mean percentage that voted democratic compared with cities without a FTSR. Democratic voters tend to strongly support policies that promote public health, such as FTSRs and other tobacco control policies. Although our analysis focused on California, these findings can be used to raise awareness of these disparities in other jurisdictions that plan to implement similar policies.

A limitation of this analysis is that we did not consider FTSRs in unincorporated counties in California. By just including cities, this analysis covered 83% of the California population (32,763,161 included in this analysis / 39,283,497 total population). Another limitation is that we did not categorize the policies based on their exemptions. Future research could use the FTSR classification system recently developed by the Truth Initiative (Donovan et al., 2021). Last, the ALA tobacco control score included one “bonus” point for having an FTSR. However, we used the 2018 ALA tobacco control score, before most cities had an FTSR, and one point would not substantially change the results as the mean score in cities with an FTSR (6.9) was much higher compared with cities without an FTSR (2.6).

Although almost 60% of California adults support a law that would prohibit the sale of all flavored tobacco products, our results show that only 20% of the California population was covered by an FTSR (California Department of Public Health, California Tobacco Control Program, 2021). A state-wide ban, SB 793, is pending for voters’ approval and would further ensure that everyone, especially youth, in California has limited access and exposure to flavored tobacco products and its associated marketing, which may eventually help reduce tobacco use among young people. Policymakers from the localities without a history of supporting tobacco control measures could focus on creating and passing tobacco control policies, such as FTSRs, to protect the health of the citizens in their jurisdictions.

## Supplemental Material

sj-docx-1-hpp-10.1177_15248399221136861 – Supplemental material for Demographics, Tobacco Use, and Tobacco Control Measures of California Cities With Flavored Tobacco Sales Restrictions Supplemental material, sj-docx-1-hpp-10.1177_15248399221136861 for Demographics, Tobacco Use, and Tobacco Control Measures of California Cities With Flavored Tobacco Sales Restrictions by Melanie S. Dove, Shichen Zheng, Sheila Pakdaman and Julia Chen-Sankey in Health Promotion Practice
