# Supplementary material for: Demographics, Tobacco Use, and Tobacco Control Measures of California Cities With Flavored Tobacco Sales Restrictions
Source: Health Promot Pract. 2022 Nov 23;25(3):455–9. doi: 10.1177/15248399221136861 (PMC10203053; doi:10.1177/15248399221136861)
Supplement: sj-docx-1-hpp-10.1177_15248399221136861 – Supplemental material for Demographics, Tobacco Use, and Tobacco Control Measures of California Cities With Flavored Tobacco Sales Restrictions [file sj-docx-1-hpp-10.1177_15248399221136861.docx]

Supplemental Table 1. List of 93 California Cities with Flavored Tobacco Sales Restrictions passed before September 2021

| Adelanto |
| --- |
| Alameda |
| Albany |
| Alhambra |
| Alturas |
| Anderson |
| Arroyo Grande |
| Auburn |
| Benicia |
| Berkeley |
| Beverly Hills |
| Buena Park |
| Burbank |
| Burlingame |
| Calabasas |
| Capitola |
| Carpinteria |
| Carson |
| Cerritos |
| Cloverdale |
| Compton |
| Corte Madera |
| Cudahy |
| Culver City |
| Cupertino |
| Danville |
| Davis |
| Delano |
| Dublin |
| East Palo Alto |
| El Cerrito |
| El Monte |
| Encinitas |
| Fairfax |
| Fremont |
| Guadalupe |
| Half Moon Bay |
| Hayward |
| Hermosa Beach |
| Imperial Beach |
| Lafayette |
| Laguna Niguel |
| Larkspur |
| Livermore |
| Long Beach |
| Loomis |
| Los Altos |
| Los Gatos |
| Mammoth Lakes |
| Manhattan Beach |
| Maywood |
| Menlo Park |
| Morgan Hill |
| Morro Bay |
| Novato |
| Oakland |
| Oroville |
| Oxnard |
| Pacific Grove |
| Palmdale |
| Palo Alto |
| Paradise |
| Pinole |
| Pleasanton |
| Portola Valley |
| Redondo Beach |
| Richmond |
| Ross |
| Sacramento |
| San Anselmo |
| San Buenaventura (Ventura) |
| San Carlos |
| San Francisco |
| San Leandro |
| San Luis Obispo |
| San Mateo |
| San Pablo |
| San Rafael |
| Santa Clarita |
| Santa Cruz |
| Santa Maria |
| Saratoga |
| Sausalito |
| Sebastopol |
| Solana Beach |
| Sonoma |
| South San Francisco |
| Tiburon |
| Watsonville |
| West Hollywood |
| West Sacramento |
| Windsor |
| Woodland |
